# Mortality of *Candidozyma auris* Candidemia Compared with Non-*C. auris* Candidemia

**DOI:** 10.3390/jof12040234

**Published:** 2026-03-25

**Authors:** Sungsoo Park, Heesuk Kim, Kilchae Hwang, Duckjin Hong, Hyeyoung Oh

**Affiliations:** 1 Division of Medicine, Sheikh Khalifa Specialty Hospital, Ras Al Khaimah P.O. Box 6365, United Arab Emirates; sungsoo.park@sksh.ae; 2 Department of Pulmonology, Seoul National University Hospital, Seoul 03080, Republic of Korea; 3 Environmental Safety Healthcare Provider Team, Sheikh Khalifa Specialty Hospital, Ras Al Khaimah P.O. Box 6365, United Arab Emirates; 4 Department of Laboratory Medicine, Sheikh Khalifa Specialty Hospital, Ras Al Khaimah P.O. Box 6365, United Arab Emirates; 5 Department of Laboratory Medicine, Seoul National University Hospital, Seoul 03080, Republic of Korea; 6 PureLab, Sheikh Khalifa Specialty Hospital, Ras Al Khaimah P.O. Box 6365, United Arab Emirates; 7 Department of Family Medicine, Seoul National University Hospital, Seoul 03080, Republic of Korea

**Keywords:** *Candidozyma auris*, candidemia, mortality

## Abstract

*Candidozyma auris* (formerly *Candida auris*) is frequently multidrug-resistant, resulting in limited treatment options and high mortality. Comparable mortality between *C. auris* candidemia and non-*C. auris* candidemia in recent studies requires confirmation in the Middle East after adjustment for confounders. This study aimed to compare mortality rates between patients with candidemia by *C. auris* and non-*C. auris Candida* species. We retrospectively analyzed 94 cases with candidemia between January 2019 and October 2025, including *C. auris* candidemia (n = 30) and non-*C. auris* candidemia (n = 64). Inverse probability weighting was used to balance baseline confounders between groups. The primary analysis used a weighted Cox proportional hazards model. Patients in the *C. auris* group had more comorbidities, greater healthcare exposure, and longer hospital stays. Crude 30-day all-cause cumulative mortality was comparable between the *C. auris* and non-*C. auris* groups (log-rank test, *p* = 0.8). The 30-day mortality of *C. auris* candidemia was similar to that of non-*C. auris* candidemia (adjusted HR 0.40; 95% CI 0.16–1.04; *p* = 0.060). Large multicenter studies involving diverse populations across different regions are warranted to validate these findings.

## 1. Introduction

*Candidozyma auris* (formerly *Candida auris*) is an emerging pathogen that has spread across 6 continents, posing a significant global public health threat. In 2022, the World Health Organization (WHO) classified *C. auris* as a critical priority pathogen to improve the overall response to this fungal pathogen [1]. *C. auris* is commonly associated with hospital-acquired infections, particularly among immunocompromised patients, and is frequently responsible for outbreaks in healthcare settings. The pathogen is often multidrug-resistant, and the resulting limited treatment options contribute to its high mortality rate [2,3].

Reported crude mortality rates for *C. auris* infections range from 30% to 72% [4,5]. This wide variability suggests that outcomes are influenced by multiple factors, such as age, comorbidities, associated risk factors, infection severity, and clinical management. Mortality rates also vary considerably across geographic regions [6]. Differences among *C. auris* clades and regional antifungal resistance patterns may further contribute to this variability, in addition to patient-specific risk factors, healthcare system characteristics, and differences in study methodologies.

It remains unclear whether mortality associated with invasive *C. auris* infections is higher than that associated with infections caused by non-*C. auris Candida* species [7]. Recent studies have reported similar mortality rates between *C. auris* infections and those caused by other *Candida* species [8,9,10]. National data from the United Arab Emirates (UAE) also showed comparable mortality rates between patients with *C. auris* or those with other *Candida* species, though this analysis included both colonized and infected patients [11]. Importantly, most previous studies have demonstrated crude mortality rates without adequately adjusting for confounding variables, limiting the ability to determine the true attributable mortality rate. For example, Colombian studies did not perform antifungal susceptibility tests for all isolates, with variability in the timing and selection of antifungal agents [9,10,12]. A study conducted in Pakistan reported the empirical use of amphotericin B as first-line therapy, in contrast to studies from other regions [13].

Given these limitations, findings from prior survival studies require confirmation in the Middle East, where few adjusted survival studies have been published. Studies that control key confounders, such as comorbidities, septic shock, and antifungal treatment, are needed to inform clinical decision-making, optimize treatment strategies, enhance infection control practices, and support the development of new therapeutic approaches. We aimed to compare mortality rates between patients with *C. auris* candidemia and those with non-*C. auris* candidemia.

## 2. Materials and Methods

### 2.1. Study Population

This retrospective study included 94 patients who developed candidemia caused by any *Candida* species between January 2019 and October 2025. Patients aged 18 years or older with the first episode of candidemia at our institution were included in the analysis. Patients with positive central venous catheter tip culture prior to candidemia were excluded. The study was conducted in a tertiary government hospital in the Northern Emirates with a capacity of 246 beds.

**Figure 1:**
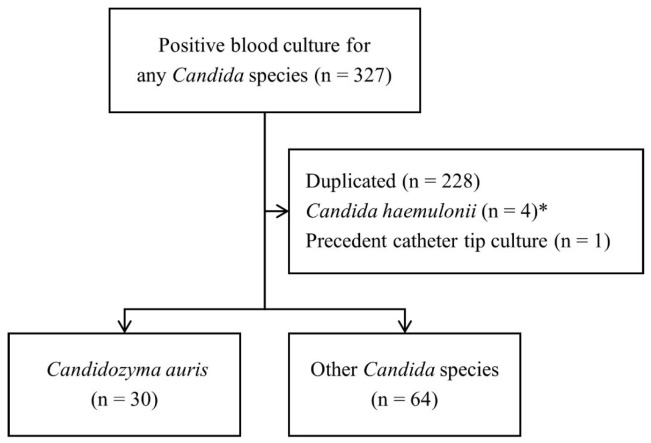
Flow diagram for case identification (n = 94). * *Candida haemulonii, Candidozyma duobushaemulonii* before 2020, and *Clavispora lusitaniae* and other *Candida* species before May 2024 were excluded.

Between 2020 and April 2024, only isolates identified as *Candida haemulonii* and *Candidozyma duobushaemulonii* (formerly *Candida duobushaemulonii*) by the VITEK2 YST card underwent further testing using VITEK MS IVD version 3.0 in our laboratory. In May 2024, we implemented VITEK MS IVD version 3.2, which accurately identifies *C. auris* and other *Candida* species. Therefore, to minimize the risk of misclassification of true *C. auris*, patients with *C. haemulonii*, *C. duobushaemulonii* before 2020, and patients with *Clavispora lusitaniae* (formerly *Candida lusitaniae*) and other *Candida* species identified by the VITEK2 YST card before May 2024 were excluded (Figure 1).

The primary outcome was 30-day all-cause mortality after the first positive blood culture for a *Candida* species. Secondary outcomes included 14-day mortality, 90-day mortality, in-hospital mortality, and length of hospital stay.

### 2.2. Data Collection

Baseline demographic characteristics and risk factors associated with *Candida* infection were extracted from electronic medical records. Demographic variables included age, sex, comorbidities, reason for admission, length of hospital stay before candidemia, location of candidemia onset, and healthcare exposure within 90 days before infection. Healthcare exposure included prior hospitalization, intensive care unit (ICU) admission, surgery, and hemodialysis. Comorbid conditions were quantified using the Charlson Comorbidity Index (CCI).

Risk factors for *Candida* infection included exposure to antimicrobial agents, immunosuppressive agents, parenteral nutrition, and vasopressors within the past month, and the presence of medical devices. Devices assessed included central venous catheters, hemodialysis catheters, arterial catheters, endotracheal or tracheostomy tubes, and urinary catheters within the past month. In addition, carriage of multidrug-resistant organisms (MDROs) and episodes of bacteremia within the past month were recorded. MDROs included extended-spectrum β-lactamase-producing bacteria, carbapenem-resistant *Enterobacterales*, carbapenem-resistant *Acinetobacter baumannii*, multidrug-resistant *Pseudomonas aeruginosa*, methicillin-resistant *Staphylococcus aureus*, and vancomycin-resistant *Enterococcus*.

To assess disease severity at candidemia onset, Sequential Organ Failure Assessment (SOFA) scores were calculated. The highest SOFA score between the day of the first positive blood culture (day 0) and the subsequent day (day 1) was used. Data regarding the presumed source of candidemia, early source control measures, such as central venous catheter removal and abscess drainage, and early or appropriate antifungal therapy were also collected. Early source control was defined as intervention within 48 h of the first positive blood culture [14]. Early antifungal therapy was defined as initiation of an antifungal agent within 24 h of the first positive blood culture [15], whereas appropriate antifungal therapy was defined as administration of an antifungal agent in accordance with antifungal susceptibility results. Finally, mortality outcomes and corresponding dates of death or last follow-up were recorded.

### 2.3. Candida Testing and Reporting

Colonies from positive bacterial culture in clinical specimens were Gram-stained, and specimens demonstrating yeast were subcultured onto Sabouraud dextrose agar (SDA) and incubated at 37 °C for 48 h. Isolates identification was performed using either matrix-assisted laser desorption/ionization-time of flight (MALDI-TOF) mass spectrometry (VITEK MS, bioMérieux, Marcy-l’Étoile, France) or VITEK AST-YS08 Test Card on the VITEK 2 (bioMérieux, Marcy-l’Étoile, France), utilizing the most recent software of the VITEK 2 identification system (version 9.02) per the manufacturer’s instructions.

Between 2020 and April 2024, only isolates identified as *C. haemulonii* and *C. duobushaemulonii* by the VITEK2 YST card underwent further testing using VITEK MS IVD version 3.0 in our laboratory. Before May 2024, isolates identified as *C. lusitaniae* and other *Candida* species by the VITEK2 YST card were reported without additional confirmation testing, potentially including undetected *C. auris* cases. From May 2024 onward, VITEK MS IVD version 3.2 was implemented, enabling accurate identification of *C. auris* and other *Candida* species.

### 2.4. Statistical Analysis

Demographic characteristics, risk factors associated with *Candida* infection, characteristics of candidemia, clinical management, and mortality outcomes were compared between patients with *C. auris* and those with non-*C. auris Candida* species. Categorical variables were compared using the chi-square (χ^2^) test or Fisher’s exact test, as appropriate, whereas continuous variables were compared using the Mann–Whitney U test. Fourteen-day, 30-day, 90-day, and in-hospital cumulative mortality were compared between the two groups using Kaplan–Meier survival analysis and the log-rank test.

Inverse probability weighting was employed to balance baseline confounders between *C. auris* and non-*C. auris* groups. A propensity score representing the probability of *C. auris* candidemia was estimated using logistic regression, including nine potential confounders (sex, location of candidemia onset, prior hospitalization, ICU admission history, surgery history, exposure to antifungal agent, corticosteroids use, CCI, and MDRO carriage). Age, diabetes mellitus, and hemodialysis were not included separately, as they are already incorporated in the CCI.

Overlap weights (average treatment effect in the overlap population) were applied rather than conventional inverse probability weights, as preliminary analysis indicated limited group overlap. However, the events-per-variable (EPV) ratio of the propensity score model was 3.3 (30 *C. auris* patients, 9 confounders), which is acknowledged as a limitation. Finally, a weighted Cox proportional hazards model with *Candida* species, early source control, and early antifungal therapy as outcome predictors was used to evaluate differences in survival between the *C. auris* and non-*C. auris* groups. Results were reported as hazard ratios (HRs) and 95% confidence intervals (CIs).

Statistical significance is set at two-sided *p* < 0.05. Statistical analyses were conducted in R statistical software (version 4.4.3), using WeightIt (version 1.5.1), cobalt (version 4.6.2), and survival packages (version 3.8-6).

## 3. Results

### 3.1. Characteristics of the Study Population

Of the 94 patients who developed any candidemia, the median age was 76.0 years (interquartile range [IQR] 62.3, 85.0), and females were 40 (42.6%) (Table 1). Sepsis or septic shock was the most common reason for admission. The median length of hospital stay before the onset of candidemia was 23.5 days ([IQR] 12.0, 51.0), and approximately 80% of cases developed candidemia in the ICU.

Patients with *C. auris* candidemia had a longer duration of hospitalization and more healthcare exposure compared with those with candidemia caused by other *Candida* species. The *C. auris* group had a higher prevalence of diabetes mellitus and higher CCI scores. Exposure to antifungal agents and corticosteroids was more frequent in the *C. auris* group. Individuals with *C. auris* candidemia were more likely to carry multidrug-resistant organisms.

### 3.2. Candidemia and Outcomes

In the non-*C. auris* group, most common *Candida* species were *C. albicans* (n = 21, 32.8%) and *C. tropicalis* (n = 21, 32.8%), followed by *C. parapsilosis* (n = 12, 18.8%) (Table S1). Of 30 cases of *C. auris* candidemia, 23 (76.7%) were resistant to fluconazole, whereas all isolates remained sensitive to caspofungin.

The most common source of infection was a central venous catheter (n = 46, 48.9%), followed by the gastrointestinal tract (n = 14, 14.9%), with similar distributions in both groups (*p* = 0.546) (Table 2). There was no difference in SOFA scores between the *C. auris* and non-*C. auris* groups (*p* = 0.567). Early source control and early antifungal therapy were achieved in 52.1% and 36.2% of patients, respectively.

**Figure 2:**
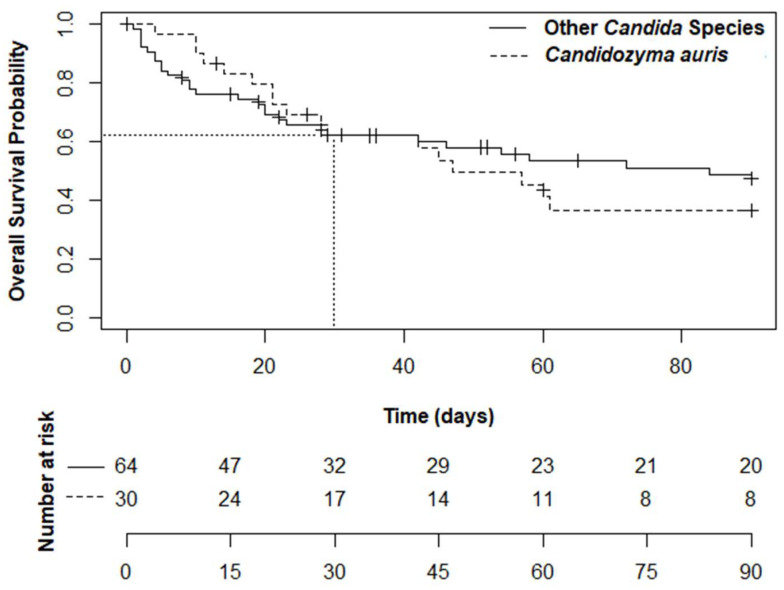
Cumulative survival following candidemia by *Candida* species.

Kaplan–Meier survival analysis demonstrated similar crude 30-day cumulative mortality between the *C. auris* and non-*C. auris* groups (38.0% vs. 37.8%; log-rank test, *p* = 0.8) (Figure 2). Fourteen-day and 90-day mortality were similar between the two groups (16.8% vs. 23.9%, *p* = 0.3; 63.2% vs. 53.8%, *p* = 0.7). In-hospital mortality was also comparable (63.3% vs. 51.6%, *p* = 0.7). Patients in the *C. auris* group tended to have longer hospital stays (32.0 vs. 22.0 days, *p* = 0.064).

In the weighted Cox proportional hazards model, the 30-day mortality of *C. auris* candidemia was similar to that of non-*C. auris* candidemia (HR 0.50, 95% CI 0.20, 1.26, *p* = 0.14) (Table 3). When early source control and early antifungal therapy were added to the model as outcome predictors, *C. auris* candidemia was associated with a trend toward lower 30-day mortality compared to non-*C. auris* candidemia (adjusted HR 0.40, 95% CI 0.16–1.04, *p* = 0.06), which did not reach statistical significance.

## 4. Discussion

Our study demonstrated that *C. auris* candidemia may not be associated with higher mortality than non-*C. auris* species. Patients with *C. auris* candidemia were older, had more diabetes, and higher CCI scores, with more healthcare exposure. The *C. auris* group also received more antifungal agents and corticosteroids, carried more MDROs, and had longer hospital stays. These findings are consistent with previous studies, indicating our study population shares similar characteristics with other reported cohorts [7]. Antifungal susceptibility testing of *C. auris* isolates showed high resistance to fluconazole and susceptibility to echinocandins, consistent with the South Asia clade (clade I), the most prevalent clade in the Middle East [16]. Crude 30-day mortality in our cohort was approximately 38%, which is comparable to the report from Pakistan [13], but lower than that from Turkey [17].

An unexpected finding in our study was that *C. auris* was the most common cause of candidemia (32%). This aligns, in part, with *a* previous report indicating that non-*albicans Candida* species are now as common as, or more prevalent than, *C. albicans* [18]. Recent literature reviews up to April 2025 highlighted the rapid global spread of *C. auris*, now reported in over forty countries, with a marked increase in prevalence over the past five years [19]. National data from the UAE also indicated a rapid rise in *C. auris* cases among all *Candida* species cases [11]. This trend may be explained by *C. auris*’s potential for widespread transmission, persistent skin colonization [20], increased use of fluconazole [17], and the impact of the COVID-19 pandemic [21].

*C. auris* can form biofilms that are resistant to antifungal penetration, contributing to colonization and resistance to multiple antifungal agents [22]. Several studies have demonstrated that C *auris* can evade neutrophil attacks and the innate immune response more effectively than *C. albicans* [23,24]. However, unlike *C. albicans*, *C. auris* generally does not form hyphae in the human body, a structure critical for tissue invasion [25]. Despite these conflicting resistance and virulence factors of *C. auris*, accumulating evidence suggests that the mortality of *C. auris* candidemia may be comparable to that of non-*C. auris* candidemia. High mortality in *C. auris* candidemia reported in previous studies may be influenced by patients’ comorbidities, infection severity, and adequacy of management [26]. Therefore, future reports of candidemia mortality should account for these confounding factors rather than relying solely on crude mortality.

Since CCI and SOFA scores are difficult to modify at the time of candidemia, early source control and timely initiation of appropriate antifungal therapy are critical for patient prognosis [27,28,29]. However, in our study, early source control and early antifungal therapy were only achieved in 30–50% of patients. This underscores the urgent need for robust antifungal stewardship programs to improve outcomes of patients with candidemia [30,31].

Early source control and early appropriate antifungal therapy were associated with a trend toward lower mortality in our cohort without statistical significance [17,32]. This may be explained by the small number of patients, the inclusion of patients who benefit less from early source control, and the influence of other confounding factors, such as infection severity [28]. The decreased hazard ratio of *C. auris* after adjusting these variables suggests that early aggressive management may attenuate the true effect of the species.

The strength of this study lies in its comprehensive inclusion of key factors influencing mortality in patients with candidemia, including source control, antifungal therapy, CCI, and SOFA scores, and the availability of antifungal susceptibility data of all *Candida* species. Previous reports from the UAE have compared crude mortality between *C. auris* and non-*C. auris* candidemia [11,33]. However, this is the first UAE report that comprehensively adjusted for confounders affecting mortality in patients with candidemia. The consistent hazard ratios of *C. auris* after inclusion of therapy variables support the robustness of our study.

Limitations of our study include the small number of cases, resulting in insufficient statistical power to detect a significant difference in *Candida* species-related mortality. The low EPV ratio (3.3) in the propensity score model raises concerns of overfitting, which may lead to unreliable propensity score estimates despite the application of overlap weighting. Changes in *Candida* species identification methods over the study period may have resulted in potential underrepresentation of true *C. auris* candidemia in earlier study years. Also, all-cause mortality was used rather than attributable mortality to candidemia. Variation in candidemia management practices precluded inclusion of follow-up culture results and recurrent infections in the analyses [34]. Large multicenter studies with diverse populations in other regions are needed to confirm our findings and to account for the wide range of confounding factors that may influence mortality.

## 5. Conclusions

The 30-day mortality of *C. auris* candidemia was similar to that of non-*C. auris* candidemia. Further large multicenter studies with diverse populations in other regions are needed to confirm these findings.

## Figures and Tables

**Table 1:** Basic demographics and risk factors associated with *Candida* infection.

	Total (N = 94)	Non-*Candidozyma auris* Group (N = 64)	*Candidozyma auris* Group (N = 30)	*p* Value
Age, median (IQR), years	76.0 (62.3, 85.0)	74.0 (55.0, 81.3)	81.0 (73.3, 90.0)	0.003
Female	40 (42.6%)	24 (37.5%)	16 (53.3%)	0.182
Reason for admission				0.360
Sepsis/Septic shock	32 (34.0%)	20 (37.5%)	12 (40%)	
Respiratory failure	24 (25.5%)	15 (23.4%)	9 (30%)	
Others	38 (40.4%)	29 (45.3%)	9 (40%)	
Days of hospitalization before candidemia, median (IQR)	23.5 (12.0, 51.8)	17.5 (9.8, 30.3)	63.5 (35.3, 123)	<0.001
ICU stay at candidemia onset	75 (79.8%)	49 (76.6%)	26 (86.7%)	0.409
Healthcare exposure within the past 90 days				
Prior hospitalization	71 (75.5%)	43 (67.2%)	28 (93.3%)	0.005
Prior ICU admission	39 (41.5%)	21 (32.8%)	18 (60%)	0.015
Surgery	20 (21.3%)	8 (12.5%)	12 (40%)	0.006
Hemodialysis	16 (17.0%)	4 (6.25%)	12 (40%)	<0.001
Comorbidities				
Diabetes mellitus	56 (59.6%)	33 (51.6%)	23 (76.7%)	0.025
Chronic kidney disease	38 (40.4%)	22 (34.4%)	16 (53.3%)	0.114
Cerebrovascular accident	24 (25.5%)	13 (20.3%)	11 (36.7%)	0.127
Cancer	27 (28.7%)	21 (32.8%)	6 (20%)	0.231
Charlson comorbidity index, median (IQR)	7.0 (5.0, 9.0)	6.0 (4.0, 9.0)	8.0 (7.0, 10.0)	0.019
Medications				
Antibiotics	86 (91.5%)	58 (90.6%)	28 (93.3%)	1.0
Antifungal agent	23 (24.5%)	8 (12.5%)	15 (50%)	<0.001
Corticosteroids *	25 (26.6%)	6 (9.4%)	19 (63.3%)	<0.001
Non-corticosteroid immunosuppressive agent	13 (13.8%)	9 (14.1%)	4 (13.3%)	1.0
Parenteral nutrition	40 (42.5%)	29 (45.3%)	11 (36.7%)	0.505
Vasopressor	60 (63.8%)	38 (59.4%)	22 (73.3%)	0.164
Tubes and catheters				
Central venous catheter	75 (83.3%)	51 (82.3%)	24 (85.7%)	0.769
Hemodialysis catheter	38 (40.4%)	25 (39.1%)	13 (43.3%)	0.822
Arterial catheter	70 (74.5%)	46 (71.9%)	24 (80%)	0.457
Endotracheal or tracheostomy tube	68 (73.9%)	42 (67.7%)	26 (86.7%)	0.076
Urinary catheter	76 (80.9%)	49 (76.6%)	27 (90%)	0.251
Microbiologic testing				
MDRO carriage	31 (33.0%)	16 (25%)	15 (50%)	0.020
Concomitant bacteremia	21 (22.7%)	11 (17.2%)	10 (33.3%)	0.111

Abbreviations: IQR = interquartile range; ICU = intensive care unit; MDRO = multidrug-resistant organism. * Corticosteroids: prednisolone 20 mg or higher dose equivalent for more than 2 weeks.

**Table 2:** Characteristics, management, and outcomes of candidemia.

	Total (N = 94)	Non-*Candidozyma auris* Group (N = 64)	*Candidozyma auris* Group (N = 30)	*p* Value
Source of infection				0.546
Central venous catheter	46 (48.9%)	31(48.4%)	15 (50.0%)	
Gastrointestinal tract	14 (14.9%)	12 (18.8%)	2 (6.7%)	
Urinary tract	4 (4.3%)	3 (4.7%)	1 (3.3%)	
Skin and soft tissue	3 (3.2%)	2 (3.1%)	1 (3.3%)	
Others	27 (28.7%)	16 (25.0%)	11 (36.7%)	
SOFA scores, median (IQR)	10.0 (6.0, 14.8)	10.0 (5.8, 14.0)	9.5 (6.5, 16.0)	0.567
Management of candidemia				
Early source control	49 (52.1%)	36 (56.3%)	13 (43.3%)	0.274
Early antifungal therapy	34 (36.2%)	24 (37.5%)	10 (33.3%)	0.819
Appropriate antifungal therapy	78 (83.0%)	52 (81.3%)	26 (86.7%)	0.769
Outcomes				
14-day cumulative mortality *	21.6% [95% CI 12.8–29.6%]	23.9% [95% CI 12.6–33.8%]	16.8% [95% CI 2.2–29.2%]	0.3
30-day cumulative mortality ^†^	37.9% [95% CI 26.9–47.2%]	37.8% [95 CI 24.2–48.9%]	38.0% [95% CI 17.4–53.4%]	0.8
90-day cumulative mortality ^‡^	56.0% [95% CI 44.3–66.9%]	53.8% [95% CI 38.6–65.7%]	63.2% [95% CI 38.6–78.0%]	0.7
In-hospital mortality	55.3%	51.6%	63.3%	0.7
Days of hospitalization after candidemia, median (IQR)	23.5 (10.3, 53.3)	22.0 (8.8, 51.0)	32.0 (18.0, 59.3)	0.064

Abbreviations: SOFA = sequential organ failure assessment; IQR = interquartile range; CI = confidence interval. * Three patients who were lost to follow-up before 14 days were censored. ^†^ Eleven patients who were lost to follow-up before 30 days were censored. ^‡^ Twenty patients who were lost to follow-up before 90 days were censored.

**Table 3:** Weighted Cox proportional hazards analyses of 30-day mortality risk factors in patients with candidemia.

		Crude HR (95% CI)	*p* Value	Adjusted HR (95% CI)	*p* Value
Early source control	No			reference	
	Yes			0.59 (0.24, 1.43)	0.2
Early antifungal therapy	No			reference	
	Yes			0.57 (0.21, 1.55)	0.3
*Candida* species	Non-*Candidozyma auris*	reference		reference	
	*Candidozyma auris*	0.50 (0.20, 1.26)	0.14	0.40 (0.16, 1.04)	0.060

Abbreviations: HR = hazard ratio; CI = confidence interval.

## Data Availability

The data presented in this study are available on request from the corresponding author. The data is not publicly available due to privacy restrictions.

## Supplementary Materials

The following supporting information can be downloaded at: https://www.mdpi.com/article/10.3390/jof12040234/s1. Table S1. The distribution of *Candida* species by change in *Candida* species identification methods.
