# Supplementary material for: Mortality of Candidozyma auris Candidemia Compared with Non-C. auris Candidemia
Source: J Fungi (Basel). 2026 Mar 25;12(4):234. doi: 10.3390/jof12040234 (PMC13117770; doi:10.3390/jof12040234)
Supplement: Supplementary file 1 [file jof-12-00234-s001.zip › jof-4188714-supplementary.pdf]

Table S1. The distribution of *Candida* species by change in *Candida* species identification methods

	**Before 2020** (N=5)	**January 2020 to April 2024** (N=71)	**May 2024 to Oct 2025** (N=18)	**Total** (N=94)
Candidozyma auris	0 (0%)	25 (35.2%)	5 (27.8%)	30 (31.9%)
**Non-*C. auris Candida* species**	5 (100%)	46 (64.8%)	13 (72.2%)	64 (68.1%)
Candida albicans	1 (20%)	18 (25.4%)	2 (11.1%)	21 (22.3%)
Candida tropicalis	1 (20%)	15 (21.1%)	5 (27.8%)	21 (22.3%)
Candida parapsilosis	2 (40%)	6 (8.5%)	4 (22.2%)	12 (12.8%)
Candida glabrata	1 (20%)	4 (5.6%)	2 (11.1%)	7 (7.4%)
Miscellaneous	0 (0%)	3 (4.2%)	0 (0%)	3 (3.2%)
